# Changeovers of vasoactive drug infusion pumps: impact of a quality improvement program

**DOI:** 10.1186/cc6209

**Published:** 2007-12-28

**Authors:** Laurent Argaud, Martin Cour, Olivier Martin, Marc Saint-Denis, Tristan Ferry, Agnes Goyatton, Dominique Robert

**Affiliations:** 1 Hospices Civils de Lyon, Hôpital Edouard Herriot, Department of Emergency and Medical Intensive Care, Lyon F-69003, France; 2 Hospices Civils de Lyon, Hôpital de la Croix-Rousse, Medical Intensive Care Unit, Lyon F-69004, France

## Abstract

**Background:**

Hemodynamic instability following the changeover of vasoactive infusion pump (CVIP) is a common problem in the intensive care unit. Several empiric methods are used to achieve CVIP. We hypothesized that the variation in these procedures could generate some morbidity. We sought to assess the effects of the standardization of practice, as a quality improvement program, on the CVIP-induced incidents.

**Materials and methods:**

We performed a prospective before-and-after intervention study including all adult patients with a diagnosis of cardiovascular failure who received a continuous infusion of vasoactive drugs or inotropic drugs. After a baseline preimplementation period (phase 1), a standardized 'quick change method' of CVIP using two syringe drivers was implemented in our intensive care unit (phase 2). Endpoints (rate and distribution of incidents: variations of systolic blood pressure >20 mmHg or heart rate >20 beats/min, and arrhythmias) were registered in both 3-month phases.

**Results:**

We studied a total of 913 CVIP events (phase 1, 435 events; phase 2, 478 events) from 43 patients. Patient characteristics were not significantly different among phases, with a majority of the patients having septic shock. The frequency of incidents was significantly (*P *< 0.0001) reduced in phase 2 (5.9%, *n *= 28) versus phase 1 (17.8%, *n *= 78). This effect was observed whichever catecholamine was used. More than 98% of incidents were blood pressure variations, with a similar distribution of the nature of incidents in both phases.

**Conclusion:**

The present study illustrates that adverse events are common following CVIP, and illustrates the positive impact of a quality improvement program to enhance inpatient safety related to this current process of care.

## Introduction

Circulatory failure is one of the most common organ dysfunctions in patients admitted to the intensive care unit (ICU) [1]. These patients often require administration of intravenous vasoactive or inotropic medications to optimize or support their cardiovascular function [2]. These potent agents have a very short half-life and are generally infused in high concentration with a low flow rate and a very narrow safety margin [3]. For these reasons, high-precision infusion pumps are recommended to maintain a precise and constant flow rate in order to achieve both constant plasma concentrations and effects on the cardiovascular system [4]. Moreover, the limited syringe capacity requires changeovers on a regular basis to ensure a continuous supply of the drugs. Achieving hemodynamic stability is a major therapeutic goal for treating patients in shock states. Difficulties in maintaining the hemodynamics during and after changeovers of vasoactive infusion pumps (CVIPs) are common problems [5-7]. Indeed, if a constant flow rate cannot be ensured then serious complications, such as hemodynamic instability and/or arrhythmia, may occur.

Several procedures are commonly used to achieve these infusion exchanges, using either a single syringe driver [8] or two syringe pumps with or without an overlapping period [5,6]. Nevertheless, it is not obvious to determine which of the two CVIP managing methods is the most efficient [5-7]. There are therefore no guidelines actually available. Despite a strong demand from nurses in the ICU to standardize these healthcare procedures, in order to improve patients' safety, practices vary widely.

We hypothesized that the lack of standardization could result in a greater number of adverse events related to CVIP. The aim of the present study was to assess the influence of a quality improvement program to help reduce the number of incidents linked to this current care in the ICU.

## Materials and methods

### Initiating a quality improvement program

Following an internal audit in our institution, we identified CVIP to be a daily problem. As recommended to overcome such difficulties [9-12], we decided to prioritize a quality improvement program for CVIP in a nine-bed unit of our university-affiliated adult medical ICU. Thanks to strong motivation in the staff, we created a multidisciplinary work team with both designated nurse and medical leaderships in order to improve healthcare quality related to CVIP. Ethical approval was obtained from the local institutional review board, and the study, including patient (or family) information, was performed in accordance with the ethical standards laid down in the 1964 Declaration of Helsinki. Patients or their families gave informed consent. All nurses and physicians were informed of the study protocol. No change in hemodynamic management for cardiovascular dysfunction (including septic shock treatment) occurred in our ICU during the study period.

### Study design

#### Phase 1: environmental scan

The first prospective and observatory phase of the study was conducted over a 3-month period. All adult patients suffering from shock who received continuous infusion of catecholamines (that is, dopamine, dobutamine, norepinephrine or epinephrine) were observed according to this quality improvement program. Cardiovascular dysfunction was defined by a systolic arterial blood pressure <90 mmHg with signs of peripheral hypoperfusion despite adequate fluid resuscitation, and by a continuous infusion of vasopressor agents or inotropic agents required to maintain systolic pressure ≥90 mmHg. In accordance with our routine clinical practices, all patients who received these drugs had a central venous catheter. Catecholamines were always infused with a minimal flow rate of approximately 2 ml/hour. Patients were continuously monitored, including their invasive arterial blood pressure. The aim of this first phase was to record all the incidents (as defined below) related to the CVIP. In this phase, nurses were free to choose the method of changeover of CVIP they usually practice.

#### Phase 2: standardization of changeover of vasoactive infusion pump

The second study phase was designed to standardize procedures for CVIP and to change behaviors. Following phase 1, during a 1-month period each nurse received a 1-day training course including practical work. We chose as an effective strategy the previously described 'quick change method', using two syringe drivers, already known by the staff [5-8]. This handling consisted of loading the new infusion with a new line into a new syringe pump, and priming the line when the running infusion was about to finish. Both syringes were maintained at bed height. Nurses started the pump and chose a high flow rate until a drop of the vasoactive drug appeared on the end of the line, in order to avoid a startup delay. The nurses then programmed the pump to the same rate and setting as the previous infusion. The cap was then removed from the spare port of the three-way stopcock and connected to the new infusion pump. The nurses then turned the three-way stopcock on to the new infusion, which closed the lumen of the old infusion. Finally, the nurses had to disconnect the old infusion and put a cap on the new spare port. Only one syringe pump model (Pilot A2^®^; Fresenius Viale™, Brezin, France) was used for this study, with 50 ml syringes (BD Plastipack^®^, Octeville, France). The protocol was continuously available for consultation in the ICU.

After this training period, as in phase 1, we recorded throughout another 3-month period all incidents related to the CVIP in patients treated with catecholamines.

### Measurements

Baseline characteristics of the patients were registered: gender, age, Simplified Acute Physiology Score II, type of admission and type of shock. Data related to CVIP were also recorded: the catecholamine and its dose. The heart rate and invasive systolic blood pressure were continuously recorded 5 minutes before and throughout CVIP on a monitor (Monitor 1165A^®^; Hewlett Packard™, Louisville, KY, USA). Baseline hemodynamics were defined as the average of three measurements preceding CVIP.

CVIP-induced incidents were defined as follows: a variation of systolic blood pressure >20 mmHg if it occurs in the first 15-minute period after the changeover; a variation of heart rate >20 beats/min in the same time interval with regard to the CVIP; and the occurrence of a documented atrial or ventricular arrhythmia.

### Statistical analysis

Assuming a CVIP-related incident frequency of 15% in the control group (phase 1) based on prestudy observations, we calculated that at least 736 CVIP events would be required for the study to have 90% power to detect a 50% reduction in the relative risk with a two-sided α level of 5%. Data are expressed as counts and proportions or as the mean ± standard deviation, as appropriate. Comparisons of categorical variables were performed using a two-sided chi-square test or Fisher's exact test, as appropriate. Continuous data were compared using an unpaired Student's *t *test. Statistical significance was defined as *P *< 0.05.

## Results

We studied 43 patients: 25 patients in phase 1 and 18 patients in phase 2. Characteristics of the patients are presented in Table 1. The most frequent cause of the acute circulatory failure was septic shock in medical patients from both phases. Baseline characteristics were not significantly different between groups (Table 1). Twenty-three patients (92%) received vasopressor drugs (that is, dopamine, norepinephrine and epinephrine) in phase 1, and 10 of these patients (43%) also received dobutamine. This was not significantly different in phase 2, with 15 patients (83%) and eight patients (53%), respectively (*P *= not significant).

**Table 1:** Baseline characteristics of patients

	Phase 1 (*n *= 25)	Phase 2 (*n *= 18)	*P *value
Gender			0.60
Male	17 (68)	12 (66)	-
Female	8 (32)	6 (34)	-
Mean age	62 ± 14	60 ± 19	0.66
Type of admission			0.72
Medical	23 (92)	16 (88)	-
Surgical	2 (8)	2 (12)	-
Type of shock			0.66
Septic	22 (88)	16 (89)	-
Cardiogenic	2 (8)	2 (11)	-
Hemorrhagic	1 (4)	0 (0)	-
Simplified Acute Physiology Score II	50 ± 22	53 ± 14	0.69

Data expressed as the number (%) of patients or as the mean ± standard deviation.

From these patients, 913 CVIP events were evaluated: 435 events in phase 1 and 478 events in phase 2. The number of CVIP procedures per patient was not significantly different in phase 1 (17.4 ± 23.4) versus phase 2 (26.5 ± 22.3) (*P *= not significant). The distribution of changeovers according to the catecholamines is presented in Table 2. Even though norepinephrine and dobutamine were the main catecholamines used in both phases, the distribution of all catecholamines was significantly different (*P *< 0.05) – with more changeovers of dobutamine and dopamine in phase 2 (45% and 22%, respectively) than in phase 1 (37% and 14%, respectively), and more changeovers of norepinephrine in phase 1 (48%) than in phase 2 (32%).

**Table 2:** Catecholamine changeover-induced hemodynamic incidents

	Phase 1	Phase 2	*P *value
Dobutamine	11/162 (7)	3/214 (1)	0.006
Dopamine	21/62 (34)	10/106 (9)	<0.001
Norepinephrine	46/207 (22)	15/155 (10)	0.002
Epinephrine	0/4 (0)	0/3 (0)	-
Total	78/435 (18)	28/478 (6)	<0.0001

Data expressed as the number of incidents/number of changeovers (%).

The doses and flow rates for each inotropic/vasoactive drug are presented in Table 3. Similar profiles were observed in both phases excepted for the norepinephrine and dopamine doses, which were significantly higher in phase 2 than in phase 1. Concentrations of these two medicines were also significantly higher in phase 2 versus phase 1, with differences between both phases averaging 0.14 mg/ml and 1.06 mg/ml for norepinephrine and dopamine, respectively (*P *< 0.05). The other mean concentrations of catecholamines (that is, dobutamine and epinephrine) were not significantly different between both phases of the study.

**Table 3:** Doses and flow rates for each vasoactive drug

	Dose (μg/kg/min)			Flow rate (ml/hour)		
	
	Phase 1	Phase 2	*P *value	Phase 1	Phase 2	*P *value
Dobutamine	15.7 ± 6.2	14.6 ± 4.5	0.56	6.3 ± 2.2	5.3 ± 1.8	0.08
Dopamine	7.5 ± 5.9	9.9 ± 5.8	<0.01	4.5 ± 1.8	4.7 ± 2.0	0.53
Norepinephrine	1.8 ± 1.2	2.4 ± 1.5	<0.001	10.4 ± 5.9	11.9 ± 6.1	0.01
Epinephrine	3.5 ± 3.2	2.9 ± 0.9	0.79	13.0 ± 9.4	12.6 ± 4.0	0.95

Data expressed as the mean ± standard deviation.

The number of patients who presented at least one CVIP-related incident in phase 2 (11/18 patients, 61%) was significantly (*P *= 0.02) reduced from phase 1 (23/25 patients, 92%). We recorded a total of 106 incidents during the study: 78 incidents in phase 1 and 28 incidents in phase 2. The frequency of incidents was significantly lower in phase 2 than in phase 1 (*P *< 0.0001), at 5.9% and 17.8%, respectively. In addition, as presented in Table 2, this beneficial effect was observed for each catecholamine, including norepinephrine. As shown in Table 4, blood pressure variations were the most frequent incidents we observed. No documented arrhythmia was observed. The distribution of incidents nature did not significantly differ (*P *= 0.18) among phases (Table 3). No fatal event was related to CVIP.

**Table 4:** Nature of incidents

	Phase 1 (*n *= 78)	Phase 2 (*n *= 28)
Decrease in systolic blood pressure >20 mmHg	49 (63)	12 (43)
Increase in systolic blood pressure >20 mmHg	28 (36)	15 (54)
Decrease in heart rate >20 beats/min	1 (1)	0 (0)
Increase in heart rate >20 beats/min	0 (0)	1 (3)
Arrhythmia	0 (0)	0 (0)

Data expressed as the number (%) of incidents.

## Discussion

In the present study we demonstrate for the first time the positive effect of a quality improvement program on the incident rate related to CVIP.

CVIP in the ICU is known to be responsible for specific morbidity, including hemodynamic compromises [5-7]. The present work represents, to our knowledge, the largest survey studying the adverse cardiovascular events related to CVIP, even if the small number of patients could be considered a limiting factor. Our results showed a high rate of incidents (about 18%) before standardization of the CVIP procedure. Systolic blood pressure variations were the most frequent CVIP-induced incidents and were more often than not related to changeovers of vasoactive infusions. These findings are in agreement with a previous ICU study from the United Kingdom, showing more than 35% of adverse effects to patient blood pressure occur using three methods of epinephrine and norepinephrine infusion pump changeovers [7]. In the present survey, a wide majority of patients had septic shock and required norepinephrine and/or dopamine to sustain hemodynamics. These vasoactive drugs have adrenoreceptor-mediated actions, especially powerful vasoconstriction effects (via α-receptors) [3]. This can explain, in the setting of sepsis with a large sensitivity to vasoconstrictive therapy, the dramatic high rate of blood pressure variations we observed in our population.

Adverse events related to CVIP can usually be explained by multifactorial reasons [6]. First, the syringe pump/infusion system can generate some flow-rate variability during continuous intravenous infusions of potent drugs, which can induce sudden hemodynamic compromises in stable patients [13]. The reason for these noncontinuous behaviors has been extensively studied in recent clinical trials [14-20]. Low flow rates should lead to oscillations in blood pressure, especially when the set infusion rate is less than 1 ml/hour [14,15]. Similarly, a startup delay in fluid delivery as long as 30 minutes can be observed because of the free play of the syringe [16], or the presence of entrained air in the syringe that can considerably affect both compliance of the infusion device and of the occlusion alarm [17,18]. Moreover, a vertical displacement of the syringe should change the hydrostatic pressure and alter delivery of small infusate volumes [19,20]. For these reasons, high-precision infusion pumps are required to perform CVIP in the ICU [4].

A second reason for adverse events is that the choice of the CVIP procedure can also affect the syringe pump output. To achieve the relay, one or two syringe drivers can be used. *In vitro *evidence suggested that the double-pump practice for syringe changes provides better consistency in the dose delivery of inotropic medications [8]. In this case, either both syringe drivers run together for a period of time or the ending infusion is stopped while a new one is started. No study, however, was able to provide objective evidence as to which method of CVIP was the most suitable [5,7]. The third, and by no means the least important, issue is the lack of knowledge on the part of the operators [21,22].

We conducted the present study to assess the influence of the standardization of CVIP procedures on quality care. We took all factors influencing the safety of CVIP, mentioned above, into account. Particularly, according to our clinical practices, we used high-precision syringe pumps and low-compliance infusion devices. We also used drug concentrations that obtain constant flow rates over 2 ml/hour, and maintained all syringe drivers at the same height. We chose the well known (even so empiric) 'quick change method' using two syringe drivers as an effective strategy to standardize our CVIP practices. Indeed, it appeared that this procedure had at least three advantages: 'the quick change method' minimized the zero-infusion time using two pumps, was very quick and simple, and was less time-consuming for nurses.

Thanks to this quality improvement program in the ICU, by changing staff behaviors, the intervention reduced the occurrence of adverse events by about 67%. We observed a similar distribution of incidents in both phases of the study with a wide majority of blood pressure variations. This dramatic patient safety improvement was effective whichever catecholamine was used, including vasoactive drugs.

Our positive results are all the more sound since doses of catecholamines related to most incidents (that is, norepinephrine and dopamine) are significantly higher in the second phase of our study. After standardization, we still observed 6% CIVP-induced incidents. We can speculate that part of these incidents, related to devices and/or imperfections of the method, cannot be cut down in the setting of critically ill patients. It could be interesting, however, to compare the positive results we obtained using the 'quick change method' with those we could expect from new expensive smart pumps, with a modular design, assisted by an internal computer, which can allow automatic relays [23]. Further research is therefore needed to refine the methods and to identify the most cost-effective means of improving CVIP. Be that as it may, the present study already provides some evidence to sustain a tremendous effort to both educate medical staff and develop clinical guidelines regarding the proper management of CVIP. In the future, it will also be important to assess, on a regular basis, the preservation of our positive results, in agreement with the required methodology of a continuous quality improvement program [9-11,24,25].

## Conclusion

In summary, the present study illustrates the high morbidity rate related to CVIP in the ICU, and provides some evidence to standardize these risky procedures to improve inpatient safety. The study emphasizes also the necessity in the future for a continuous quality improvement program, including nurses in interdisciplinary teamwork, into general use of all ICU healthcare practices.

## Key messages

• Hemodynamic instability following CVIP is a common problem in the ICU.

• Without guidelines currently available, several empiric methods are commonly used to achieve changeovers of syringes.

• The present study illustrates the high rate of blood pressure variations associated with the lack of standardization of these procedures.

• Implementation of a standardized method, based on clinical evidence and local resources, could dramatically reduce adverse events linked to this practice.

## Abbreviations

CVIP = changeover of vasoactive infusion pump; ICU = intensive care unit.

## Competing interests

The authors declare that they have no competing interests.

## Authors' contributions

LA conceived of the study, participated in its design and coordination, helped to draft the manuscript and performed the statistical analysis. MC participated in the analysis and interpretation of data and helped to draft the manuscript. OM, MS-D, TF and AG participated in the design of the study, in nurse training for the protocol and in the acquisition of data. DR coordinated the study, and was involved in revising the manuscript critically. All authors read and approved the final version of the manuscript.
